# Evaluation of cine imaging during multileaf collimator and gantry motion for real‐time magnetic resonance guided radiation therapy

**DOI:** 10.1002/acm2.13085

**Published:** 2020-11-23

**Authors:** Jerrold E. Kielbasa, Sanford L. Meeks, Patrick Kelly, Twyla R. Willoughby, Omar Zeidan, Amish P. Shah

**Affiliations:** ^1^ Department of Radiation Oncology Orlando Health – UF Health Cancer Center Orlando FL USA

**Keywords:** cine MRI, MRgRT, percent image uniformity, spatial distortion

## Abstract

**Purpose:**

Real‐time magnetic resonance guided radiation therapy (MRgRT) uses 2D cine imaging for target tracking. This work evaluates the percent image uniformity (PIU) and spatial integrity of cine images in the presence of multileaf collimator (MLC) and gantry motion in order to simulate sliding window and volumetric modulated arc therapy (VMAT) conditions.

**Methods:**

Percent image uniformity and spatial integrity of cine images were measured (1) during MLC motion, (2) as a function of static gantry position, and (3) during gantry rotation. PIU was calculated according to the ACR MRI Quality Control Manual. Spatial integrity was evaluated by measuring the geometric distortion of 16 measured marker positions (10 cm or 15.225 cm from isocenter).

**Results:**

The PIU of cine images did not vary by more than 1% from static linac conditions during MLC motion and did not vary by more than 3% during gantry rotation. Banding artifacts were present during gantry rotation. The geometric distortion in the cine images was less than 0.88 mm for all points measured throughout MLC motion. For all static gantry positions, the geometric distortion was less than 0.88 mm at 10 cm from isocenter and less than 1.4 mm at 15.225 cm from isocenter. During gantry rotation, the geometric distortion remained less than 0.92 mm at 10 cm from isocenter and less than 1.60 mm at 15.225 cm from isocenter.

**Conclusion:**

During MLC motion, cine images maintained adequate PIU, and the geometric distortion of points within 15.225 cm from isocenter was less than the 1 mm threshold necessary for real‐time target tracking and gating. During gantry rotation, PIU was negatively affected by banding artifacts, and spatial integrity was only maintained within 10 cm from isocenter. Future work should investigate the effects imaging artifacts have on real‐time target tracking during MRgRT.

## 1 INTRODUCTION

The ViewRay MRIdian (ViewRay Inc, Oakwood Village, OH, USA) system integrates a magnetic resonance imaging (MRI) unit with a linear accelerator (linac) to deliver MR‐guided radiation therapy (MRgRT). The use of MRgRT allows tumors to be visualized in real time during treatment with excellent soft tissue differentiation and no additional exposure to ionizing radiation. Gating of the beam based on tracking of internal anatomy during treatment spares normal tissue and ensures the tumor is not underdosed due to patient motion. Without MRgRT, an additional margin is added to the clinical target volume to account for internal motion during treatment. This internal target volume (ITV) ensures that the tumor reliably receives the appropriate dose, but also results in greater normal tissue irradiation.

There are currently several technological challenges that result from the integration of a linac with an MRI system.^1^ Both systems interfere with each other, and a review of the relevant interactions can be found in Ref. 1. The focus of this work is on the effects linac component motion have on MR image quality. The motion of the multileaf collimator (MLC) and gantry produce eddy currents, field inhomogeneities, and nonlinear gradients. Each of these can result in geometric distortions that affect the spatial integrity of the image.^2^, ^3^ Additionally, eddy currents produce banding artifacts that interfere with the signal intensity. Deviations in signal intensity that are not related to patient anatomy can result in target tracking errors.^1^, ^4^, ^5^, ^6^ For these reasons, real‐time MRgRT systems use step‐and‐shoot IMRT, in which the gantry and MLC are static during delivery, to maintain image quality during treatment. As a consequence, the advantages that sliding window IMRT and volumetric modulated arc therapy (VMAT) have over step‐and‐shoot delivery, specifically plan quality and treatment time, are not currently available in MRgRT.

Both sliding window IMRT and VMAT allow more freedom to modulate beams for conformal shaping of the dose distribution than is available in step‐and‐shoot IMRT.^7^ Each has been shown to produce treatment plans that are dosimetrically superior to those of step‐and‐shoot IMRT, although the results are more modest for sliding window.^8^, ^9^ The dosimetric improvements include better dose homogeneity, PTV coverage, and OAR sparing. This has, in part, been used as an argument for VMAT being a better choice than MRgRT for certain treatments such as lung SBRT.^10^

A second advantage sliding window IMRT and VMAT have over step‐and‐shoot delivery is reduced treatment time.^8^ Faster treatments have the benefits of increasing throughput (so that more patients can benefit from the treatment) and decreasing discomfort (from, e.g., positional strain or a full bladder). The issue of patient comfort is arguably even more important during MRgRT because patient discomfort can lead to patient motion, which in turn can lead to gating‐based treatment interruptions. Under these circumstances, patients who have trouble tolerating treatment may have difficulty completing a fraction.^11^

If an MRgRT system could move from step‐and‐shoot to sliding window, or even volumetric modulated arc therapy (VMAT) delivery, the results would be improved plan quality and decreased treatment time. For sliding window to be implemented, the MR image quality would have to be maintained during MLC motion. Furthermore, the implementation of VMAT would require that image quality be maintained during both MLC and gantry motion. In this work, we evaluate the spatial integrity and percent image uniformity (PIU) of the 2D cine images used for tracking and gating during MRgRT in the presence of MLC and gantry motion. Cine images are not held to the same standard (as defined by the American College of Radiology (ACR) tests) typically established for higher resolution 3D imaging sequences. Since that is the case, this study evaluated the 2D cine images against a baseline of treatment conditions, i.e. static gantry and static MLC.

Previous investigations of the spatial integrity of ViewRay’s 2D cine images have been done under static gantry and MLC conditions.^2^, ^5^ Hu et al^5^ whose procedures we adapted to our experimental conditions, found a maximum deviation of 1.1 mm for points 10 cm from isocenter. Ginn et al^2^ developed a software to analyze the distortion for all points that do not lie in an artifact region. For markers lying within 10 cm of isocenter, they measured a mean distortion to be 0.51 mm, and a maximum distortion of 1.67 mm. Neither study reported the gantry position for their measurements. This work presents the first evaluation of the ViewRay MRIdian’s 2D cine imaging in the presence of moving MLC and moving gantry. Data lying in artifact regions are included so the same measurement points can be used throughout component motion.

## 2 MATERIALS AND METHODS

### 2.A MRIdian system overview

The ViewRay MRIdian system combines a 0.35 T split bore superconducting magnet, a 6 MV flattening filter free (FFF) standing wave linear accelerator, and a fully integrated adaptive treatment planning system. The system uses a 70 cm bore and has 20 cm‐50 cm diameter spherical field of view (FOV). The radiation therapy system can deliver a dose rate of 650 cGy/min at the source‐to‐axis distance of 90 cm. A double‐stack, double‐focus MLC is used to shape the beam for either 3D conformal or step‐and‐shoot IMRT delivery. The treatment planning system calculates dose using a Monte Carlo algorithm that accounts for effects of the 0.35 T magnetic field.

Both volumetric and 2D cine imaging are available and use a balanced Steady State Free Precession (bSSFP) pulse sequence known as True Fast Imaging with Steady State Precession (TRUFI). This pulse sequence is excellent for real‐time imaging because it prevents image saturation over time, making it less sensitive to motion related artifacts.^12^, ^13^ The real‐time 2D cine imaging is done in the sagittal plane with a resolution of 3.5 × 3.5 mm^2^ at a rate of 4 frames per second (fps). The slice thickness in cine mode can be 5 mm, 7 mm, or 10 mm. The high resolution 92 s 3D volumetric scans used in this study have 35 × 35 × 43 cm^3^ FOV and an isotropic spatial resolution of 0.15 cm.

### 2.B Phantoms

**Fig. 1:**
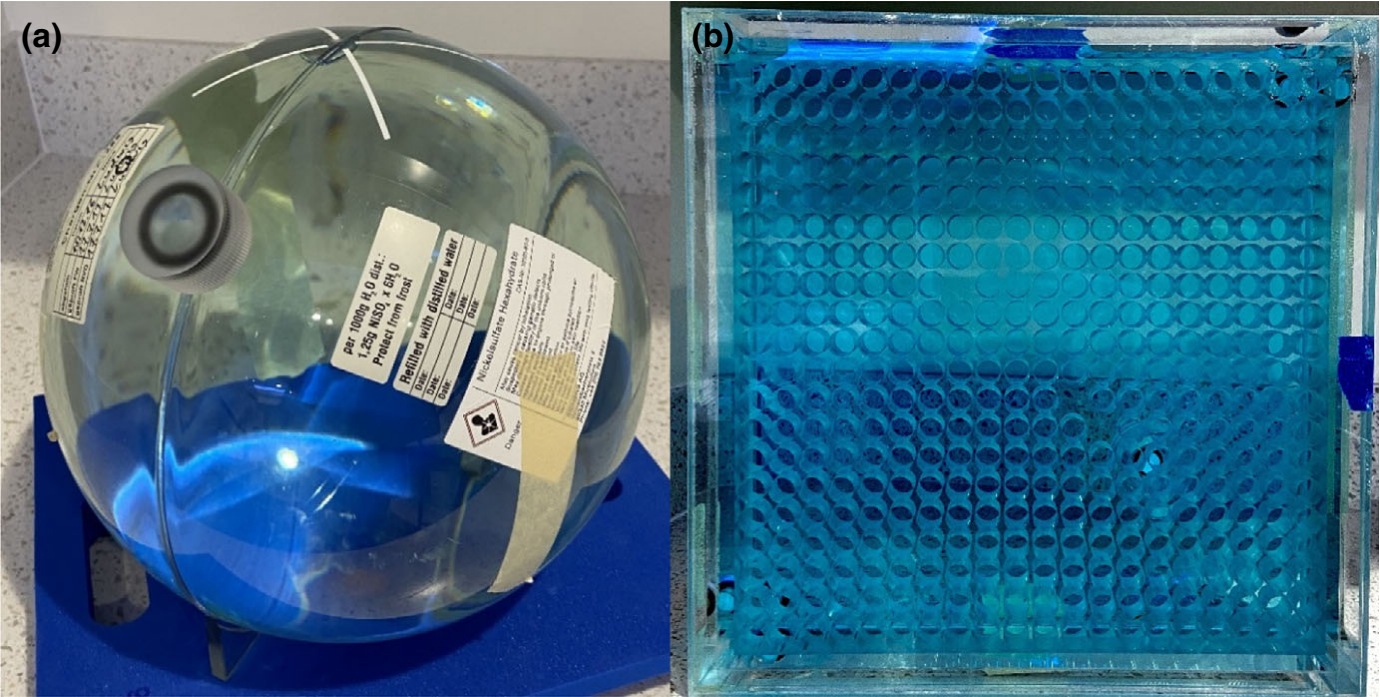
Phantoms used to characterize cine images. (a) 24 cm NEMA phantom used for percent image uniformity measurements. (b) Linear uniformity phantom used for spatial integrity measurements.

Percent image uniformity was evaluated using the 24 cm diameter spherical NEMA phantom (Siemens 4761065, Siemens Medical Solutions USA, Inc., Malvern, PA, USA) shown in Fig. 1a. The phantom is made of polymethyl methacrylate (PMMA) and contains nickelsulfate hexahydrate.

Spatial integrity was evaluated using the ViewRay‐supplied uniformity linearity phantom (Fluke 76‐907, HP Manufacturing, Cleveland, OH). The PMMA phantom is filled with an MR contrast solution (18.02 mM CuSO_4_) and contains a 288 × 288 mm^2^ grid of open cylinders (Fig. 1b). The cylinders are 14.5 mm apart, 13 mm in diameter, and have a 25 mm depth. They are arranged in a 20 × 20 grid with three missing near the center for a total of 397 cylinders.

### 2.C Experimental setup

ercent image uniformity measurements were done by imaging the 24 cm spherical NEMA phantom (Fig. 1a) at the system’s isocenter using the head and neck surface coils. Static gantry and MLC data were collected in both 3D (92 sec, 35 × 35 × 43 cm^3^ FOV) and 2D cine (35 × 35 cm^2^ FOV, 5 mm slice thickness) modes with the gantry at 330°. The 3D data were only used to evaluate the PIU for each setup, as that can vary based on coil placement. The 2D data were used as baseline data as they are representative of step‐and‐shoot delivery currently used. Moving gantry data were acquired by manually moving the gantry from 360° to 90° while imaging in cine mode.^2^ Moving MLC data were acquired by running the MLC optical‐sensor homing (MLC positional second check) while imaging in cine mode.

The static MLC and gantry spatial integrity data were collected by imaging the uniform linearity phantom (Fig. 1b) using the torso surface coils at isocenter in both 3D (92 sec, 35 × 35 × 43 cm^3^ FOV) and cine (35 × 35 cm^2^ FOV, 5 mm slice thickness) modes. The 3D data were used to establish the position of the phantom so that the geometric distortion in cine images could be evaluated. Because the magnetic susceptibility of the gantry is not uniformly distributed, the gantry position affects the homogeneity of the static magnetic field and can therefore produce geometric distortions.^2^, ^14^ For this reason, the static data were taken at multiple gantry positions in order to discriminate between geometric distortions arising from different static gantry positions and those arising from gantry motion. Based on characterization of our system using a ViewRay provided technical service report, we found that the minimum isocenter shift resulting from varying gantry positions occurs when the gantry is at 330°, the 3D static gantry data collected at 330° were used as the true phantom position when determining spatial deviation. As with the PIU measurement, the moving gantry data were collected by imaging in cine mode while the gantry was moved from 360° to 90°, and the moving MLC data were collected by imaging in cine mode while running the MLC positional second check. The uniform linearity phantom was aligned in the sagittal plane and centered with the virtual isocenter in accordance with the spatial integrity test done during monthly quality assurance (QA).^3^

### 2.D Percent image uniformity analysis

**Fig. 2:**
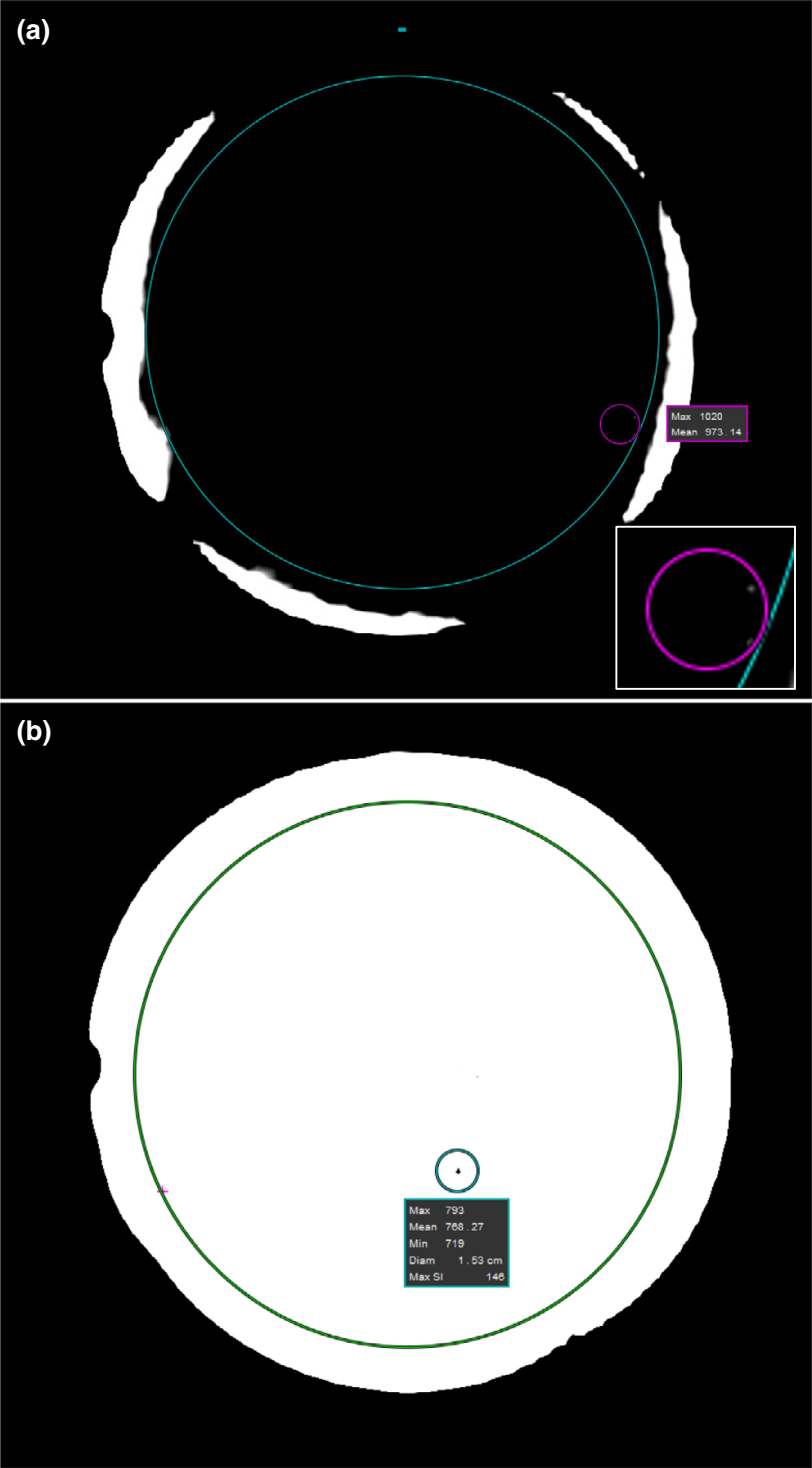
MR images of 24 cm spherical NEMA phantom used for PIU measurements. (a) Window leveling, mean signal ROI (large circle), and maximum singal ROI (smaller circle) used to find *MaxROI*. Inset shows magnification of maximum signal ROI to show the brightest pixels within the mean signal ROI. (b) Window leveling, mean signal ROI (large circle), and minimum singal ROI (smaller circle) used to find *MinROI*.

Image uniformity was evaluated by calculating the percent image uniformity.

(1) $$PIU=100\times \left(1‐\frac{MaxROI‐MinROI}{MaxROI+MinROI}\right),$$

where *MaxROI* and *MinROI* were found using the procedure outlined in the American College of Radiology MRI Quality Control Manual.^15^ Briefly, for both 3D and cine images, a 2D mean signal ROI that was concentric with the phantom and covered 75% of the phantom’s cross‐sectional area was created. The measurements to determine the *MaxROI* and the *MinROI* were taken within this area. The *MaxROI* was found by adjusting the window level so that only a small number of bright pixels were visible within the phantom (Fig. 2a). A small measurement ROI with an area that is 0.15% of the FOV area was generated to include the brightest pixels. The mean signal value within the measurement ROI was the *MaxROI*. The *MinROI* was found in the same way, except that the window level was adjusted so that only a small number of the darkest pixels were visible within the phantom (Fig 2b). The ACR recommends a PIU of 87.5% and establishes 85% as passing.^15^ However, these tolerances are established for ACR recommended sequences used for monthly QA rather than clinical scans. Therefore, the PIU of the cine images acquired during linac component motion were compared to the PIU of the cine images acquired under the static linac conditions currently used for treatment.

### 2.E Spatial integrity analysis

**Fig. 3:**
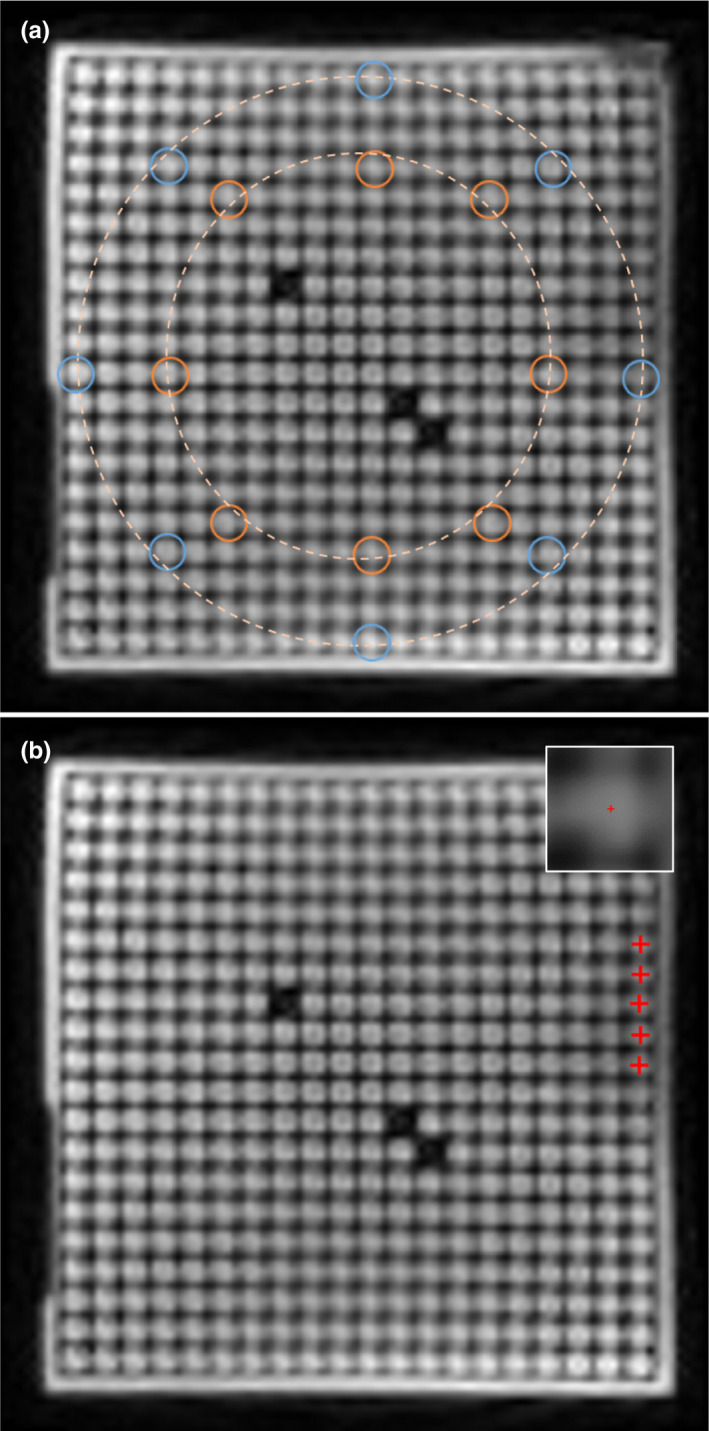
MR images of the uniform linearity phantom. (a) measurement locations 10 cm (inner circle) and 15.225 cm (outer circle) from isocenter. (b) marker localization in region of low signal due to imaging artifact (inset‐ magnification of marker in low signal region).

Spatial distortion is evaluated by determining the deviation between the marker positions in an MR image and their true positions. ViewRay provides a tool that automates this for 3D images (it is not designed to be used on 2D cine images). It is possible to automate marker localization for the 2D cine images as well, however the banding artifacts that occur during linac component motion make it particularly challenging to consistently identify makers and their positions. Therefore, the analysis of the 2D cine spatial distortion was done in accordance with the imaging characterization performed by Hu et al^5^ Measurement locations were chosen to lie on two circles centered at the isocenter with radii of 10 cm and 15.225 cm. Eight measurement locations spaced every 45° were located on each circle (Fig. 3a). The larger radius (15.225 cm) corresponds to the outermost rows and columns of markers in the phantom, making it the largest circle that will allow 8 measurement points. If a measurement location was between markers, the nearest marker was used. Hu, et al. used a larger spatial integrity phantom which allowed them to use a radius of 17.5 cm for the outer circle (note that this distance from isocenter corresponds to a change in the passing threshold from 1 mm deviation to 2 mm deviation for monthly QA). During MRgRT, the most critical tracking structures lie well within a 15 cm radius, and therefore the data collected in this study are representative of clinical situations.

In order to improve marker localization, 2D cine images were upsampled using the bicubic method from the native planar resolution (3.5 × 3.5 mm^2^) to 0.015 × 0.015 mm^2^. The center of the phantom was determined using the 3D static gantry and MLC images obtained with the gantry at 330°, where the minimum isocenter distortion occurs. The position of each marker was determined in MATLAB (The Mathworks Inc. Natick, MA, USA) using the marker’s center of mass. In order to include the same markers in each measurement, regardless of the severity and location of imaging artifacts, each marker’s position was determined after manually thresholding the image. The threshold value was empirically set so that the marker was clearly distinguished from the surrounding markers. Fig. 3b shows an example of marker position identification in a region of reduced signal intensity that is made possible by adaptive thresholding. The result of this thresholding is that the center of mass was determined using the brightest 50‐60% of each marker’s pixels. The true position of each marker was calculated using its known horizontal and vertical distances from the center of the phantom (as measured at 330°).

Each image was also rigidly registered to the 3D 330° static gantry in MIM (MIM software, Cleveland, OH) using the central most markers. Any translations of the images (which were collected on a stationary phantom) correspond to a shift in the imaging isocenter.

## 3 RESULTS

### 3.A Percent image uniformity

**Fig. 4:**
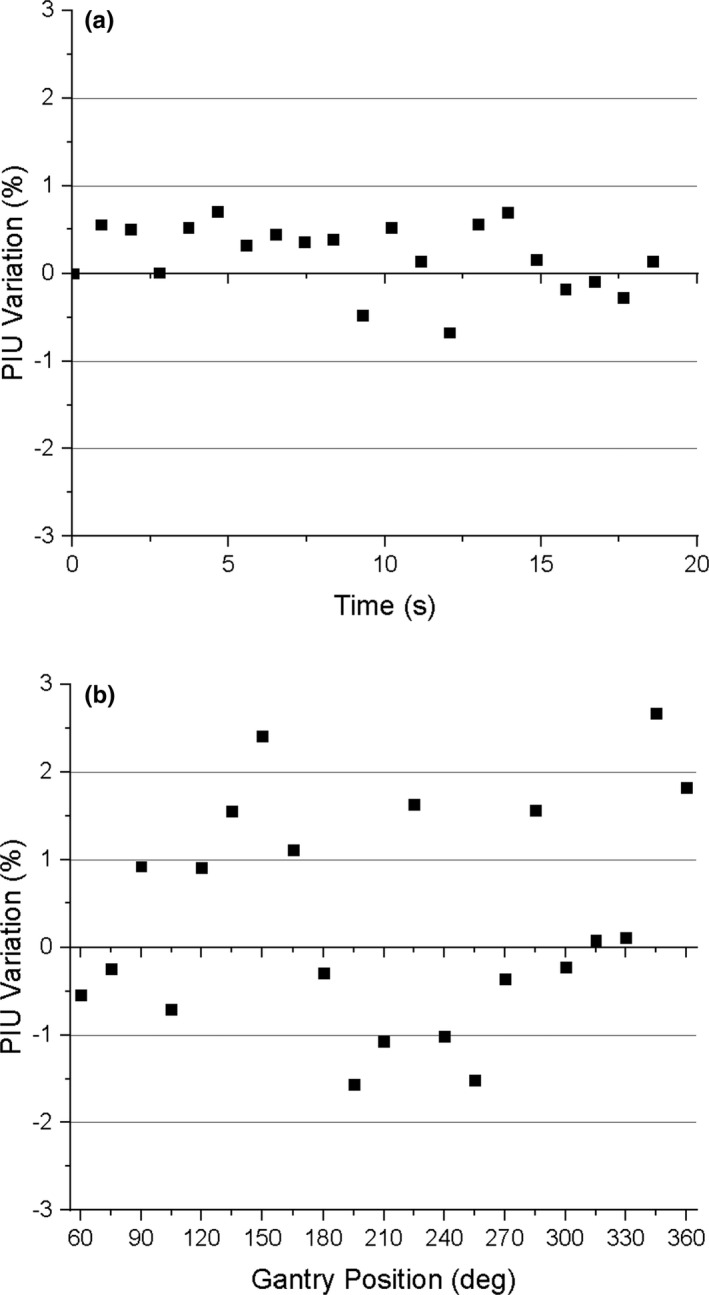
Variation of the PIU from static linac component conditions as (a) the MLC is moved and (b) the gantry is rotated from 360° to 90°.

Prior to the moving MLC test, the 3D static PIU was measured to be 88.3%, and the 2D cine static PIU was measured to be 85.6%.^4^ The variation of the PIU of the 2D cine images from the static value as the MLC leaves move is presented in Fig. 4a. It can be seen that the PIU does not vary by more than ±1% as the MLC leaves move through the MLC positional second check process. Prior to the moving gantry test, the 3D static PIU was measured to be 86.2%, and the 2D cine static PIU was measured to be 81.9%. The variation of the PIU of the 2D cine images from static conditions as the as the gantry is rotated from 360° to 90° ranges from −1.6% to 2.7% and is shown in Fig. 4b.

### 3.B Spatial integrity

#### 3.B.1 Moving MLC

**Fig. 5:**
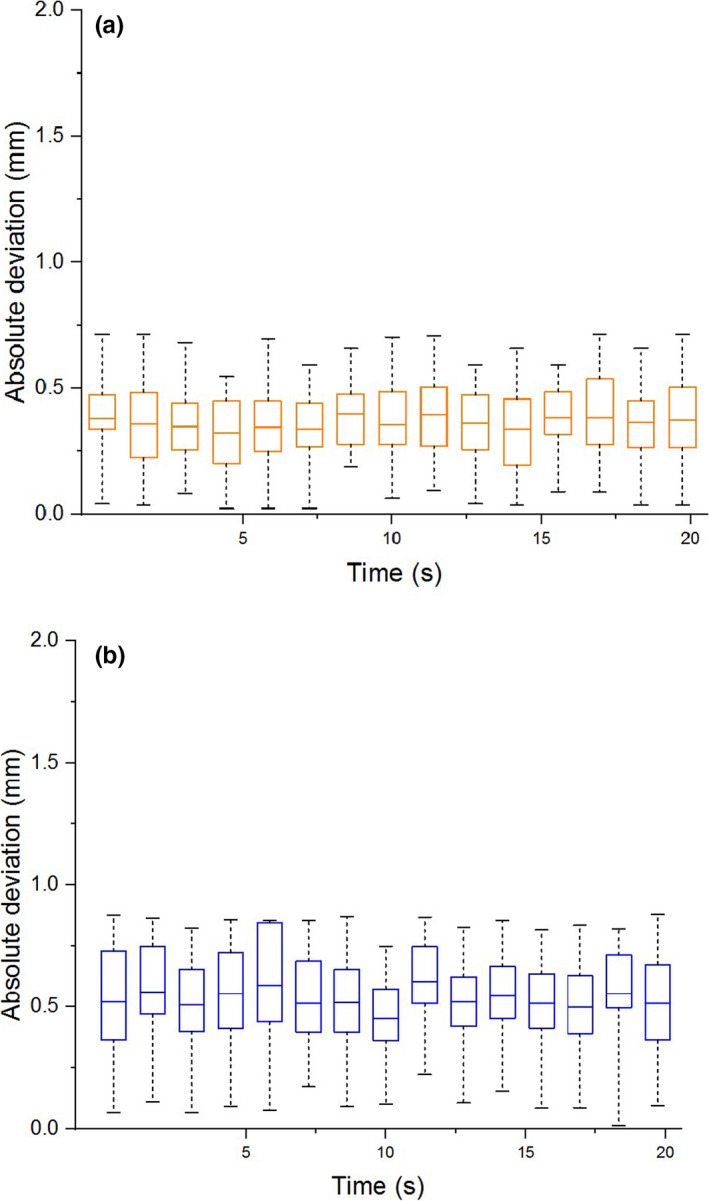
Geometric distortion of the measured position of points lying 100 mm and 152.25 mm from isocenter during MLC motion. (a) Whisker plots for absolute deviation of points 100 mm and (b) 152.25 mm from isocenter. The horizontal line within each box marks the mean deviation, the edge of each box indicates the 25th and 75th percentiles, and the whiskers show the maximum and minimum of the data.

**Fig. 6:**
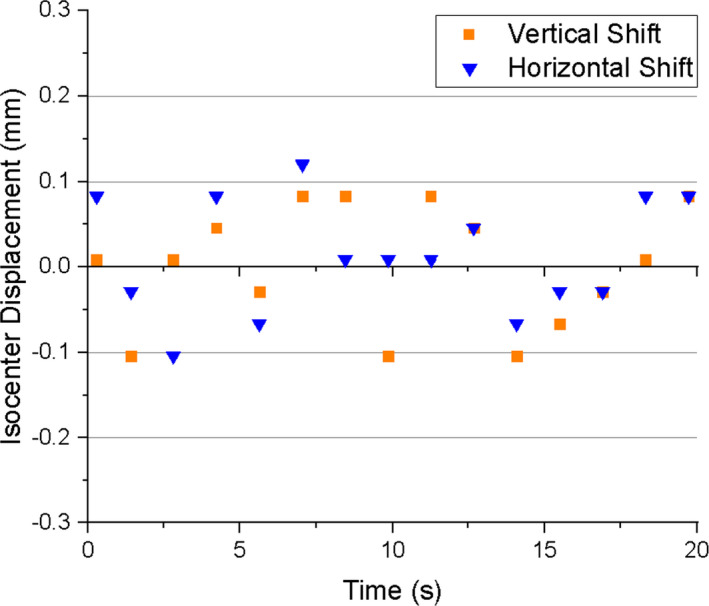
Vertical and horizontal isocenter shift in the presence of MLC motion.

Fig. 5 shows the mean geometric distortion during MLC movement for both inner (10 cm from isocenter) and outer (15.225 cm from isocenter) measurement points. The mean deviation is less than 0.45 mm for the inner points throughout the MLC motion, and the maximum deviation for those points was 0.72 mm (Fig. 5b). The mean deviation for the outer points did not exceed 0.60 mm through the MLC motion, and the maximum deviation for those points was 0.88 mm (Fig. 5c). Fig. 6 shows the displacement of the imaging isocenter, which was measured as the translational displacement from the 3D high resolution image collected at 330°. Throughout MLC motion, the isocenter deviation did not exceed 0.12 mm in both the longitudinal (Y) and vertical (Z) directions.

#### 3.B.2 Static gantry

**Fig. 7:**
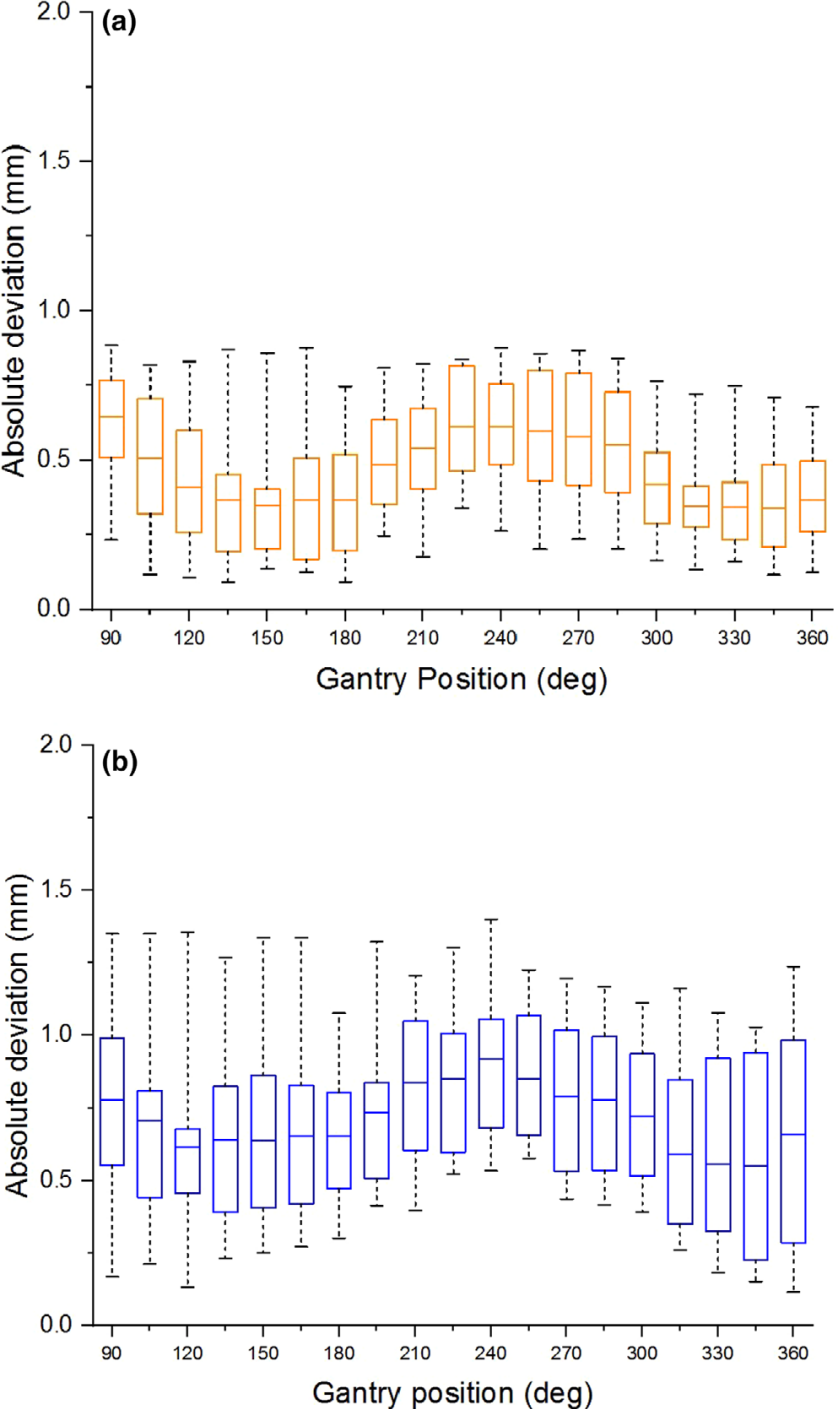
Geometric distortion as a function of static gantry position. (a) Whisker plots for absolute deviation of points 100 mm and (b) 152.25 mm from isocenter. The horizontal line within each box marks the mean deviation, the edge of each box indicates the 25th and 75th percentiles, and the whiskers show the maximum and minimum of the data.

The mean geometric distortions as a function of static gantry position for both the inner and outer measurement points are plotted in Fig. 7. The mean geometric distortion for the inner points was greatest at a gantry angle of 90°. At this position, the mean and maximum distortions were 0.65 mm and 0.88 mm (Fig. 7b), respectively. The greatest distortion for the outer points occurred at 240°, where the mean and maximum were 0.92 mm and 1.4 mm (Fig. 7c), respectively. The geometric distortion shows a gantry‐position‐dependent behavior with minima at 150° and 330°, a maximum at 240°, and apparent approach toward another maximum between 360° and 90°.

**Fig. 8:**
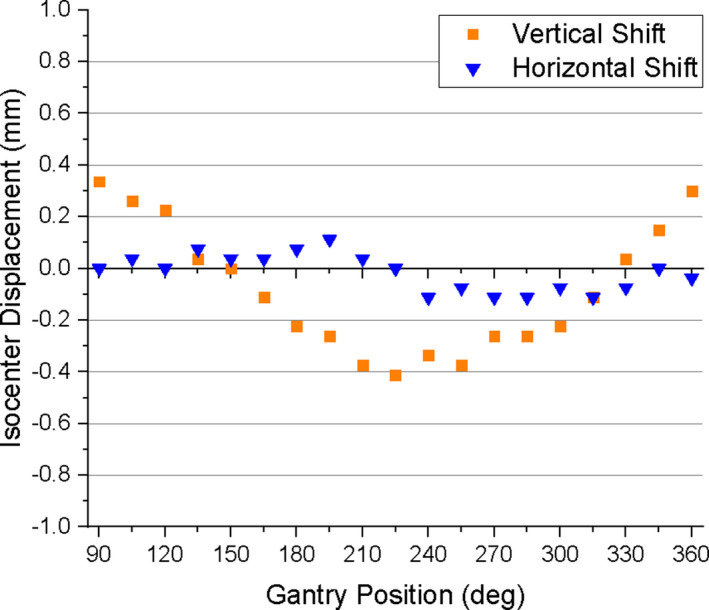
Vertical and horizontal isocenter shift as a function of static gantry position.

The shift in the imaging isocenter is shown in Fig. 8. The longitudinal (Y) isocenter displacement ranges from a maximum positive value of 0.11 mm at 195° to a maximum negative value of −0.11 mm at 285°. The vertical (Z) isocenter displacement shows a clear gantry‐position‐dependent behavior. Looking at Fig. 8, as one moves along the horizontal axis from 90° to 360° the vertical isocenter displacement starts at 0.34 mm and moves in the negative direction until reaching an inflection point at 225° (where the deviation is −0.41 mm) and then moves in the positive direction, reaching a value 0.30 mm at 360°.

#### 3.B.3 Moving gantry

**Fig. 9:**
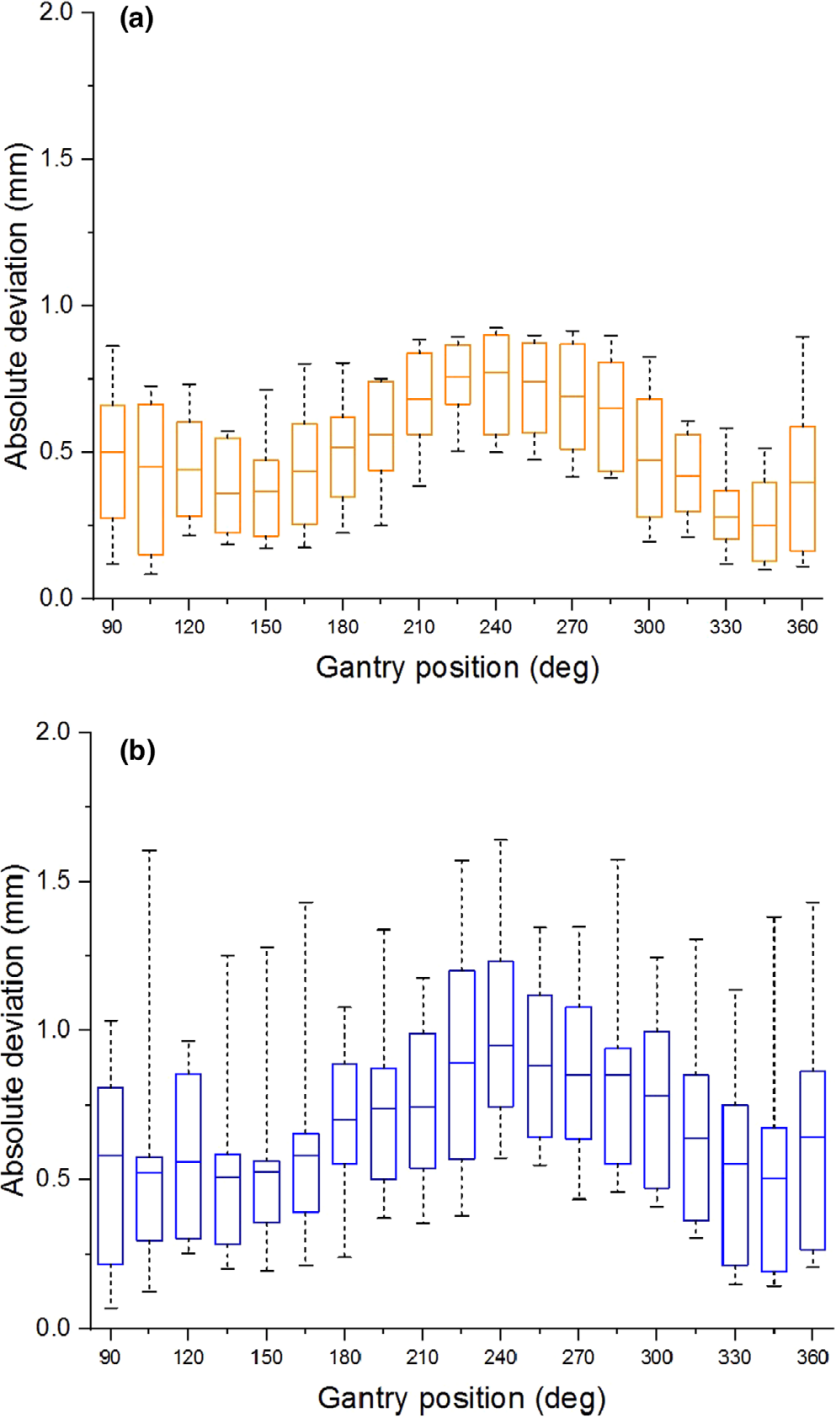
Geometric distortion as the gantry is rotated from 360° to 90°. (a) Whisker plots for absolute deviation of points 100 mm and (b) 152.25 mm from isocenter. The horizontal line within each box marks the mean deviation, the edge of each box indicates the 25th and 75th percentiles, and the whiskers show the maximum and minimum of the data.

As with the static gantry data, the mean geometric distortion as a function of moving gantry position is displayed in Fig. 9. The mean geometric distortion was greatest at a gantry angle of 240°. At this position, the mean and maximum distortions for the inner points were 0.77 mm and 0.92 mm (Fig 9b) respectively. The mean and maximum distortions for outer points were 0.95 mm and 1.6 mm (Fig. 9c), respectively. Again, the geometric distortion shows a gantry‐position‐dependent behavior. The minima are slightly displaced (135° and 345°, cf 150° and 330°). The local maximum at 240° is still present, though, as is the apparent approach toward another maximum between 360° and 90°.

**Fig. 10:**
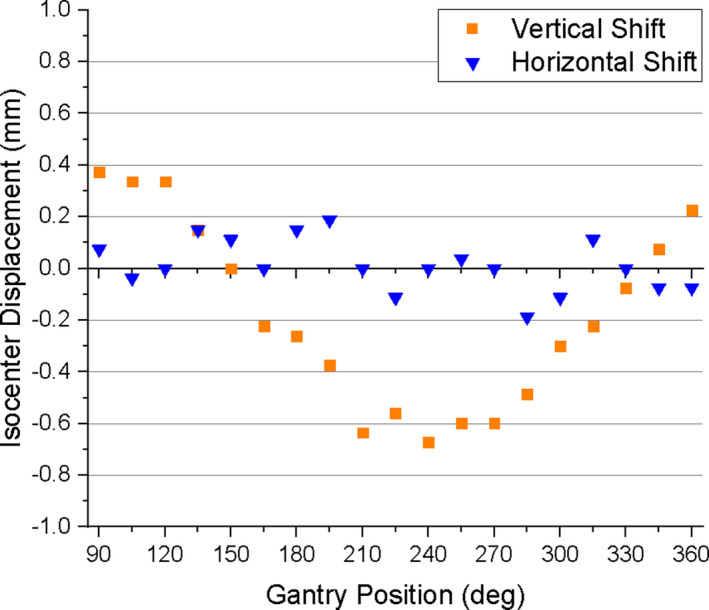
Vertical and horizontal isocenter shift as the gantry is rotated from 360° to 90°.

The shift in the imaging isocenter is shown in Fig. 10. The longitudinal (Y) isocenter displacement ranged from 0.19 mm at 195° to −0.19 mm at 285°. The vertical (Z) isocenter displacement exhibits a similar gantry position dependence as the static gantry data. Moving from 90° to 360° along the horizontal axis in Fig. 10, the displacement starts at 0.37 mm and moves in the negative Z direction until it reaches a maximum negative value of −0.67 mm at 240°, beyond which it returns toward positive displacements reaching a value of 0.22 mm at 360°.

## 4 DISCUSSION

In this work, real‐time 2D cine images obtained using the MRIdian 0.35 T MRI were characterized under the separate conditions of moving MLC and moving gantry. These conditions were chosen because they are necessary for either sliding window (moving MLC only) or VMAT (moving MLC and moving gantry) treatment delivery. Either of those delivery modes would improve plan quality and decrease treatment times compared to the step‐and‐shoot delivery currently used in MRgRT. Because 2D cine imaging is used for real‐time target tracking, these cine images would need to maintain signal uniformity and spatial integrity during MLC motion for sliding window delivery and during both MLC and gantry motion for VMAT delivery. Constancy of image uniformity is necessary so that the relative signal intensities of the target structure and surrounding tissues can be reliably used to perform real‐time tracking of the tracking target structure. High spatial integrity of the image is necessary so that real‐time tracking is performed with accurate target positioning.

The results of our image quality tests performed during MLC motion show that the PIU does not vary by more than ±1% from the static linac conditions currently used during treatment (Fig. 4a). No banding artifacts were observed that would jeopardize target tracking during treatment. The mean geometric distortion of points 10 cm from isocenter was less than 0.4 mm for every image analyzed throughout the MLC motion (Fig. 5), and the maximum deviation was 0.72 mm. The mean geometric distortion for points 15.225 cm from isocenter was less than 0.60 mm (Fig. 5), and the maximum deviation was 0.88 mm. Therefore, all geometric distortions measured within 15.225 cm from isocenter do not exceed the 1 mm threshold for acceptable spatial integrity.

**Fig. 11:**
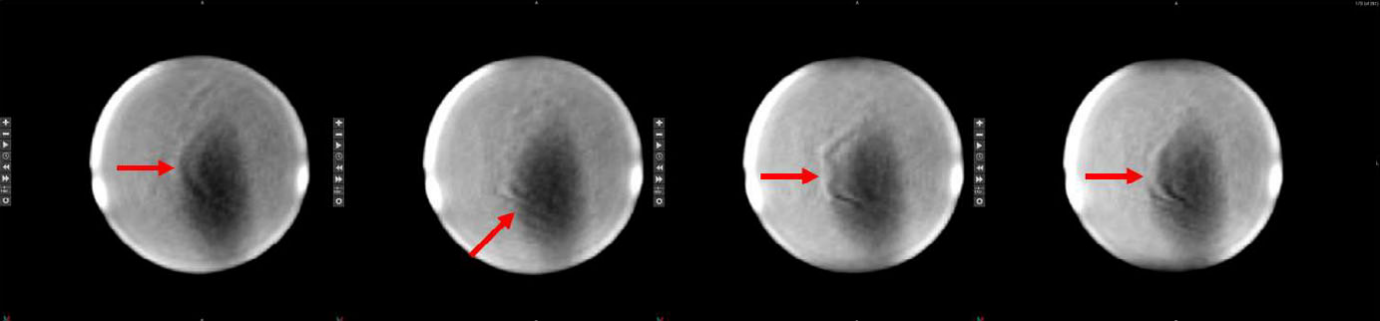
Cine MR images of the NEMA 24 cm spherical phantom as the gantry is rotated through 5°. Banding artifacts resulting from gantry motion are indicated by the red arrows..

The image quality tests performed during gantry rotation from 360° to 90° show that the PIU variation relative to static conditions ranged from −1.6% to 2.7% (Fig. 4b), and this increased variation was due in part to banding artifacts present during the movement of the gantry (Fig. 11). The signal intensity variations resulting from these artifacts could prevent reliable tracking of target structures during MRgRT.

Characterization of the effect of gantry motion on spatial integrity was more involved than in the case of moving MLC because it is not only the gantry *motion* that can affect image quality, but the static gantry position as well. The gantry does not have a uniform magnetic susceptibility, and therefore its static position may affect the magnetic field homogeneity, which in turn affects geometric distortion.^2^ Therefore geometric distortion analysis was done for both static gantry and moving gantry from 360° to 90°. The static gantry distortion had a clear dependence on gantry position in which local minima separated by 180° occurred at 150° and 330°, and a local maximum occurred at 240°. The data show that the distortion was increasing for the gantry angles beyond the measurement limitations of this experiment (i.e. between 360° and 90°). One would expect another local maximum to occur at or near 60°, as it is 180° from the observed maximum at 240° and 90° from each of the observed minima at 150° and 330°. This should, however, be verified. For the measurement points lying 10 cm from isocenter, the mean distortion was less than 0.65 mm for all gantry positions (Fig. 7), and the maximum deviation was 0.88 mm. Therefore, at 10 cm from isocenter, all deviations measured on the 2D cine image were within the 1 mm limit considered acceptable for MRgRT. For the measurement points located 15.225 cm from isocenter, the mean distortion was less than 0.92 mm for all gantry positions (Fig. 7), and the maximum deviation was 1.4 mm. While this maximum deviation exceeds the 1 mm tolerance for points within 17.5 cm, it is consistent with the conditions currently used during treatment. Measurement of the displacement of the imaging isocenter (Fig. 8) showed that the vertical (Z) component is a significant source of the gantry dependent geometric distortion, as it exhibits similar gantry dependent behavior (minima at 150° and 330°, and an absolute maximum near 240°). The magnitude of the longitudinal (Y) isocenter shift did not exceed 0.11 mm and exhibited no strong gantry‐position‐dependent behavior. The static gantry‐position‐dependent isocenter displacement of the ViewRay 3D imaging was previously investigated by Latifi et al.^14^ In their paper, the minima and maximum of the vertical isocenter displacements occurred at the same positions that we report, and the horizontal isocenter exhibited a similarly minimal dependence on gantry position.

**Fig. 12:**
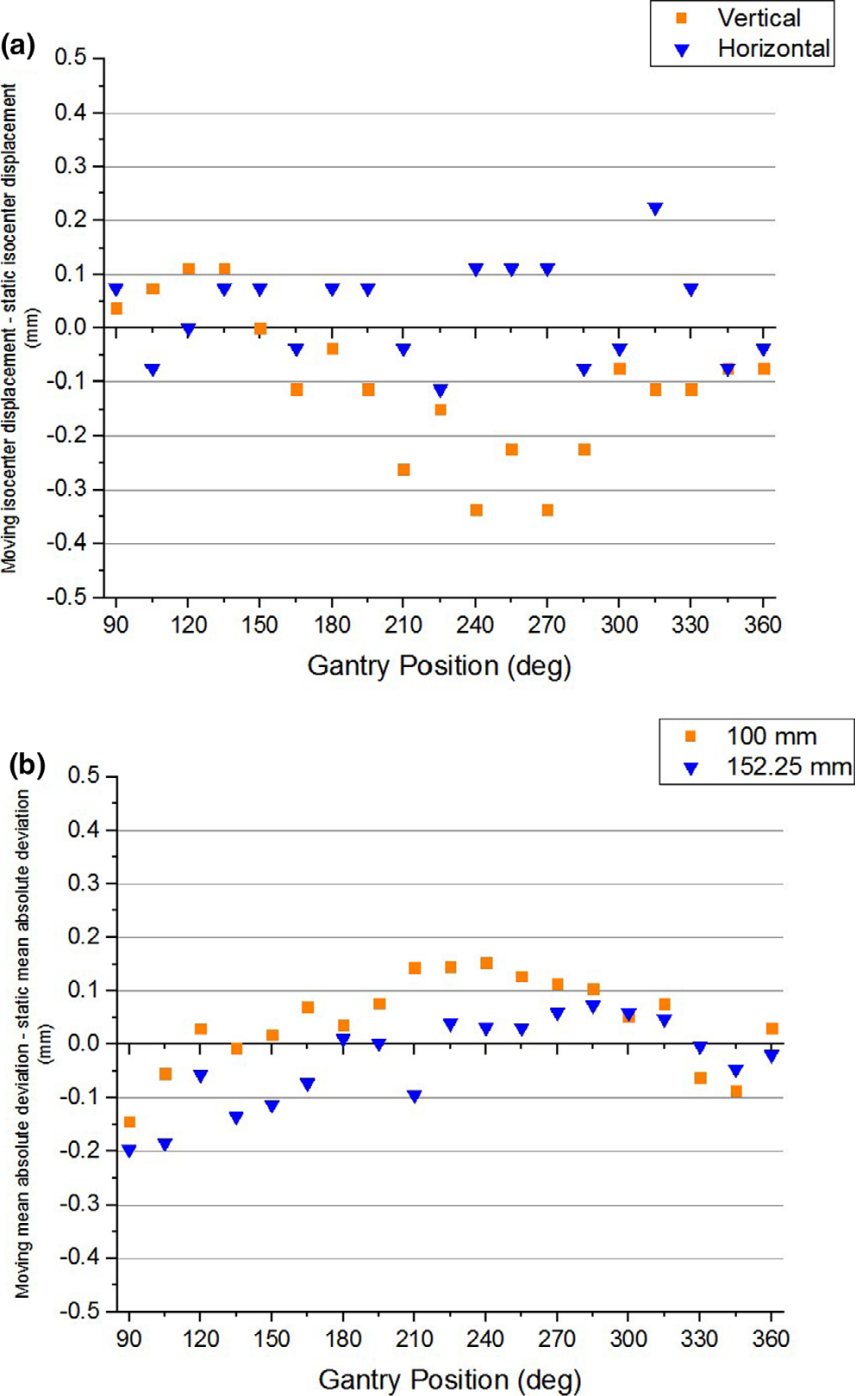
Difference between moving and static gantry dependent (a) isocenter displacement and (b) mean absolute error as a function of gantry position.

With the static gantry‐position‐dependent distortion measured, the moving gantry distortion data could be better understood. The geometric distortion exhibited a similar gantry position dependence, with a local maximum at 240° and local minima near 150° and 330° (Fig. 9). The maximum geometric distortions under moving gantry conditions were slightly greater than those of static gantry. The greatest mean distortion and the maximum distortion values for points 10 cm from isocenter were 0.77 mm and 0.92 mm, respectively (cf. 0.65 mm and 0.88 mm for static gantry). It should be noted that all deviations for the inner measurement points were still within the 1 mm threshold. The greatest mean distortion and maximum distortion for the markers lying 15.225 cm from isocenter were 0.95 mm and 1.6 mm respectively (cf. 0.92 mm and 1.4 mm for static gantry). As with the static gantry data, the maximum deviation at 15.225 cm from isocenter exceeds the 1 mm tolerance. In order to illustrate the effects that gantry motion alone has on both isocenter displacement and geometric distortion, the static gantry data were subtracted from the moving gantry data for each (Fig. 12). In Fig. 12a it can be seen that subtracting the static gantry‐position‐dependent isocenter displacement from the moving gantry‐position‐dependent isocenter displacement results in a vertical displacement of less than 0.4 mm and a horizontal displacement of less than 0.25 mm for all gantry positions. Fig. 12b shows the results of subtracting the static gantry mean absolute deviation from the moving gantry mean absolute deviation. The moving gantry data, both at 100 mm from isocenter and at 152.25 mm from isocenter, were within 0.2 mm of the static gantry data for all gantry positions.

The image distortions discussed in this paper only represent those that result from the ViewRay system. During cine imaging and target tracking, additional patient‐induced distortions also arise.^3^, ^16^, ^17^ These are caused by chemical shifts, variations in magnetic susceptibility, and patient movement. The effects these distortions have on cine tracking will need to be investigated, and those results would need to be incorporated into any evaluation of the accuracy of cine target tracking.

This work presents the first evaluation of the ViewRay MRIdian’s 2D cine imaging in the presence of moving MLC and moving gantry. The effect of moving MLC on PIU was not appreciable, and the cine images could potentially be used for target tracking. The geometric distortions that were present during MLC motion at distances within 15.225 cm from isocenter were all less than the 1 mm threshold for MRgRT. The effect of moving gantry increased the PIU variability due to banding artifacts that could prevent target tracking. The effect of moving gantry on spatial integrity was not substantially different from the distortion arising from static gantry position, which is currently used for treatment. Within the 10 cm radius, the distortion was within the 1 mm tolerance. Most target tracking is done on structures within 10 cm of the isocenter, therefore the most significant obstacle to VMAT MRgRT is issue of banding artifacts that occur during gantry motion. There have been successful banding artifact correction algorithms developed.^18^, ^19^ If corrections could be implemented in real‐time, the image quality could be maintained through gantry motion making VMAT MRgRT with real‐time tracking a possibility.

## 5 CONCLUSIONS

In this work, the spatial integrity and PIU of the 2D cine imaging of the MRIdian MRgRT system that is used for motion monitoring and gating has been characterized under the conditions of moving MLC and moving gantry. The procedures used were adopted from the ACR MR Quality Control Manual and from Hu et al.^5^ During MLC movement, the PIU and spatial integrity within 15.225 cm from isocenter were adequate for cine target tracking and gating. There is a gantry‐position‐dependent geometric distortion that is present whether the gantry is in motion or static. This distortion results in some points exceeding the 1 mm threshold for spatial integrity for points lying 15.225 cm from isocenter. However, points lying 10 cm from isocenter maintain acceptable sub‐millimeter spatial integrity through gantry rotation from 360° to 90°. Gantry motion does not appear to create substantially greater geometric distortions than those due to static gantry position. The PIU during gantry motion is negatively affected by banding artifacts to the point that target tracking may be unreliable. This data cannot be used to draw conclusions about VMAT delivery because (1) MLC and gantry motion were tested independently and (2) there was no test of target tracking in the presence of the observed banding artifacts. Further work could characterize the effect of banding artifacts on the ability of the real‐time tracking system to deform the tracking structure within a compromised image‐quality environment. This should include an analysis of the spatial behavior of the banding artifacts to determine if there is any consistency in their location that would allow systematic correction. Additionally, the effects of simultaneous MLC and gantry motion, as well as patient‐induced artifacts, should be evaluated.

## CONFLICT OF INTEREST

No conflicts of interest.

## AUTHOR CONTRIBUTIONS

Jerrold E. Kielbasa collected the data, designed the analysis, performed the analysis, interpreted the results, and drafted the manuscript. Sanford L. Meeks conceived the study, interpreted the results, and contributed to the writing of the manuscript. Patrick Kelly interpreted the results and contributed to the writing of the manuscript. Twyla R. Willoughby interpreted the results and contributed to the writing of the manuscript. Omar Zeidan interpreted the results and contributed to the writing of the manuscript. Amish P. Shah conceived of the study, designed the experiment, collected the data, interpreted the results, and contributed to the writing of the manuscript. All authors approved the manuscript.

## Notes

^1^ It is worth noting that there are patient‐specific sources of image distortion, namely chemical shifts and magnetic susceptibility, that cannot be evaluated with phantom studies.^2^

^2^ We were determined to collect data for ≈ 270 s of continuous acquisition. This corresponded to ≈ 270° of gantry rotation. Since ViewRay imaging is subject to a gantry limit switch, we chose 360°‐90° as the range of gantry rotation for these measurements.

^3^ While our monthly quality assurance (QA) procedure evaluates spatial integrity in all three anatomical planes, only the sagittal plane is relevant to 2D cine imaging.

^4^ Note that the ROIs used to calculate PIU are taken within a volume of uniform magnetic susceptibility. Therefore, a higher contrast, but less noisy, 3D scan sequence may have a higher PIU than a lower contrast, but noisier, 2D cine sequence.
